# RNA-Sequencing Analysis of the Viral Community in Yellow Catfish (*Pelteobagrus fulvidraco*) in the Upper Reaches of the Yangtze River

**DOI:** 10.3390/ani14233386

**Published:** 2024-11-25

**Authors:** Wenzhi Liu, Huiwu Tian, Jie Ma, Mingyang Xue, Yong Zhou, Mengmeng Li, Jingwen Jiang, Yuding Fan, Mingdian Liu

**Affiliations:** 1 Yangtze River Fisheries Research Institute, Chinese Academy of Fishery Sciences, Wuhan 430223, China; liuwenzhialisa@yfi.ac.cn (W.L.); tianhw@yfi.ac.cn (H.T.); majie_shou@163.com (J.M.); xmy@yfi.ac.cn (M.X.); zhouy@yfi.ac.cn (Y.Z.); lmm20000312@163.com (M.L.); j178381682@outlook.com (J.J.); 2 Graduate School, Chinese Academy of Agricultural Sciences, Beijing 100081, China; 3 College of Fisheries and Life Sciences, Shanghai Ocean University, Shanghai 201306, China

**Keywords:** Yangtze River, yellow catfish, *Pelteobagrus fulvidraco*, viral community, RNA sequencing, genetic diversity

## Abstract

Different viruses are abundant in aquatic ecosystems. There has been limited research on the viral communities in the upper reaches of the Yangtze River. Yellow catfish (*Pelteobagrus fulvidraco*), an important economic fish that is widely distributed in the upper reaches of the Yangtze River, was selected as the research object. Using RNA sequencing, we identified 11 viruses belonging to the *Adintoviridae*, *Tombusviridae*, *Caudovirales*, *Microviridae*, *Picornavirales*, and other bacteriophage families. The predominant viral families/order in Luzhou (LZ), Fuling (FL), and Wanzhou (WZ) were *Caudovirales*, *Adinoviridae,* and *Microviridae*, respectively. The virome from WZ had a unique community composition, with a high abundance of *Picornavirales* compared with LZ and FL. In LZ, the predominant double-stranded RNA virus family was *Siphoviridae*. Phylogenetic analyses showed that viruses presented high genetic diversity. Phylogenetically, Wenling pleuronectiformes picornavirus was close to the family *Caliciviridae*, which includes yellow catfish calicivirus (YcCV), responsible for the massive mortality of yellow catfish in 2020. This study provides insights into the viral community composition in yellow catfish in the upper reaches of the Yangtze River, revealing a diverse and unique river water virome and providing clues for future research on the origin of viral pathogens.

## Simple Summary

We investigated the viral community in yellow catfish (*Pelteobagrus fulvidraco*) using RNA-sequencing technology across three locations (Fuling, Luzhou, and Wanzhou) in the upper reaches of the Yangtze River. Eleven viral species were identified, comprising four double-stranded DNA viruses, two single-stranded DNA viruses, and five single-stranded RNA viruses. A significant number of phage sequences were detected at these three sampling sites, representing families such as *Siphoviridae*, *Myoviridae*, *Microviridae*, and other phage families, most of which belonged to the order *Caudovirales*. Notably, the virome derived from Wanzhou exhibited a distinct pattern in the composition of its viral community, characterized by a high abundance of the order *Picornavirales*. Furthermore, *Adinoviridae* was identified as the predominant viral family in Fuling. Our findings give new perspectives on viral diversity and transmission dynamics in yellow catfish.

## 1. Introduction

Viruses constitute a substantial factor contributing to the morbidity and mortality of aquatic lifeforms [1,2,3]; therefore, understanding viral habitats and diversity is essential to develop strategies for prevention and control and to prepare for emergency outbreaks [4,5]. Numerous known and novel viruses have been identified in marine waters, lakes, sewage, and ballast water, revealing the viral composition and distribution [6,7,8,9]. However, exploration of viral diversity in rivers has been limited, despite their importance for the sustainable management of water resources [10]. Additionally, the aquatic ecological environment influences both intraspecific and interspecific viral transmission in aquatic animals [11,12]. Therefore, investigating the distribution and characteristics of viruses across different regions of a river is crucial.

The Yangtze River, Asia’s longest and the world’s third-longest river, is a crucial water resource that supports the livelihoods of millions of people [13]. Its upper reaches extend from Galadandong on the Qinghai–Tibet Plateau to Yichang in Hubei Province, covering 4511 km [14]. This section traverses six provinces and municipalities: Qinghai, Tibet, Sichuan, Yunnan, Chongqing, and Hubei [14]. Sichuan and Chongqing, in particular, are notable for their dense populations and advanced economic development, making them prime locations to study river water viromes [15]. However, the viral community composition in these regions remains largely unexplored. 

In the present study, we investigated the fish resources in Sichuan (Luzhou City) and Chongqing (Fuling City and Wanzhou City) in the upper reaches of the Yangtze River in 2023 and identified several fish species common to all three locations, notably silver carp (*Hypophthalmichthys molitrix*), yellow catfish (*Pelteobagrus fulvidraco*), and common carp (*Cyprinus carpio*). Among them, yellow catfish, a teleost fish from the family *Bagridae*, has emerged as a key species in these three regions [16,17]. This species is widely distributed across China, Japan, South Korea, and other parts of East and South Asia [16]. In recent years, the rapid expansion of yellow catfish aquaculture has led to a significant decline in their population because of microbial diseases, caused by viruses, parasites, and bacteria, resulting in substantial economic losses [18,19,20,21,22]. Additionally, the parental stock of yellow catfish primarily consists of wild specimens collected from the Yangtze River Basin or adjacent lake areas for breeding purposes [23]. This raises concerns about the potential transmission of viral pathogens from these wild parents to cultured populations. Therefore, investigating the viral community in yellow catfish in the upper reaches of the Yangtze River might offer valuable insights into the origins of viral diseases affecting cultured stocks. Furthermore, as a bottom-dwelling omnivorous fish, yellow catfish mainly consumes zooplankton larvae and aquatic insects during its juvenile stage. However, the adults feed on small fish and invertebrates [24]. This species is currently used to monitor environmental pollution because of its prominence in Chinese ecosystems [25]. Therefore, we selected yellow catfish as the primary research object to monitor the viral community in the upper reaches of the Yangtze River.

The objective of this study was to comprehensively investigate the viral community of yellow catfish from Sichuan and Chongqing Cities in the upper reaches of the Yangtze River. Therefore, our primary focus was on yellow catfish sampled in LZ, FL, and WZ. Following RNA sequencing to identify the viruses present in yellow catfish, we conducted phylogenetic analyses to assess the genetic diversity of major virus groups based on viral hallmark genes. Our findings present a viral distribution pattern in the river water ecosystem, which will be useful to guide the sustainable management and utilization of freshwater resources.

## 2. Materials and Methods 

### 2.1. Study Area and Sample Collection

This study was conducted from May to August 2023 in the upper reaches of the Yangtze River, specifically targeting Sichuan (Luzhou (LZ) City) and Chongqing (Fuling (FL) and Wanzhou (WZ) Cities), China. Nine kidney samples were collected from yellow catfish across three areas within three cities. This sampling section spanned approximately 450 km, representing about one-tenth of the length of the upper reaches of the Yangtze River. Detailed information on the sampling sites is provided in Table 1. To minimize microbial variation within the same region, kidney samples were collected from three different yellow catfish in each area. These samples were immediately stored in liquid nitrogen and expediently conveyed to the laboratory for the RNA-seq analysis.

### 2.2. Nucleic Acid Extraction 

Total RNA was isolated from kidney tissues of yellow catfish using a TRIzol reagent kit (Invitrogen, Carlsbad, CA, USA) in accordance with the manufacturer’s instructions. The quantity of the isolated RNA was determined using an Agilent 2100 Bioanalyzer (Agilent Technologies, Palo Alto, CA, USA), and its quality was evaluated using RNase-free agarose gel electrophoresis.

### 2.3. RNA-Sequencing (RNA-Seq) Analysis

Total RNA was subjected to a modified RNA-seq library preparation protocol, adapted from Liu et al. [18]. Initially, ribosomal RNAs (rRNAs) were removed from the extracted total RNA, leaving messenger RNAs (mRNAs) and non-coding RNAs (ncRNAs). The isolated RNAs were fragmented using the supplied fragmentation buffer, producing short fragments suitable for reverse transcription into complementary DNAs (cDNAs) using random primers. A reaction mixture containing RNase H, DNA polymerase I, dNTPs (with dUTP replacing dTTP), and a buffer were utilized to synthesize second-strand cDNA. The resulting cDNA fragments were purified using a QIAquick PCR purification kit (Qiagen, Venlo, The Netherlands), after which end-repair was performed, poly(A) tails were added, and ligation with Illumina sequencing adapters (Illumina, San Diego, CA, USA) was carried out. Subsequently, the second-strand cDNA was subjected to digestion by uracil-N-glycosylase (UNG). The digested products underwent size selection via agarose gel electrophoresis afterward by PCR amplification. Subsequent sequencing was performed by Gene Denovo Biotechnology Co. (Guangzhou, China) utilizing the Illumina HiSeq 4000 platform. Bowtie 2 (v2.0.6) was adopted to align the filtered reads against the reference genome of the yellow catfish [26], facilitating the elimination of host sequences. All sequence reads generated in this study have been deposited in the NCBI database under the accession number MZ 988401.

### 2.4. Discovery of Target Viral Sequences

To identify potential viruses in yellow catfish from the upper reaches of the Yangtze River, the remaining reads were assembled de novo using MIRA Assembler (v. 4.0.2). Contigs and distinct singletons displaying little to no similarity at the nucleotide level were analyzed for similarity using BLASTX against the GenBank protein database. Additionally, Contigs were compared with the comprehensive viral RefSeq database using BLASTX and BLASTP in NCBI. The ultimate contig annotation was carried out using Geneious (v. 9.1.3, Biomatters, Auckland, New Zealand).

### 2.5. PCR Detection of Target Viral Sequences

For the potential viruses infecting yellow catfish in the upper reaches of the Yangtze River, reverse transcription–PCR (RT-PCR)/PCR assays were employed to detect these viruses in kidney tissues. Primers were developed in accordance with the RNA-seq results (Table 2). The RT-PCR reaction was conducted utilizing an RT-PCR amplification kit (Takara, Shiga, Japan). Subsequently, the PCR reaction was performed on the cDNA samples (thirty-five cycles were carried out at 95 °C for 5 min, followed by 94 °C for 1 min, 54–58 °C for 1 min, and 72 °C for 1 min; subsequently, an extension was performed at 72 °C for an additional 10 min) to obtain PCR products with different target band sizes depending on the primer location in the different viruses. Healthy kidney tissue cDNA of yellow catfish was used as a negative control. 

### 2.6. Phylogenetic Analyses

Phylogenetic analyses were conducted on the predicted protein sequences of the viral genes discovered in this study, together with reference strain protein sequences from various virus groups sourced from the NCBI GenBank database. Conserved protein families and domains were characterized through the use of ORF Finder (https://www.ncbi.nlm.nih.gov/orffinder/, Accessed on 15 September 2024). Sequence alignments of deduced amino acid (aa) sequences were performed utilizing MEGA 7.0 (Phoenix, AZ, USA) in the MUSCLE package under the default parameters. Phylogenetic trees were created through the utilization of the maximum-likelihood method and 1000 bootstrap replicates.

## 3. Results

### 3.1. Sequencing in the Upper Reaches of the Yangtze River

**Figure 1:**
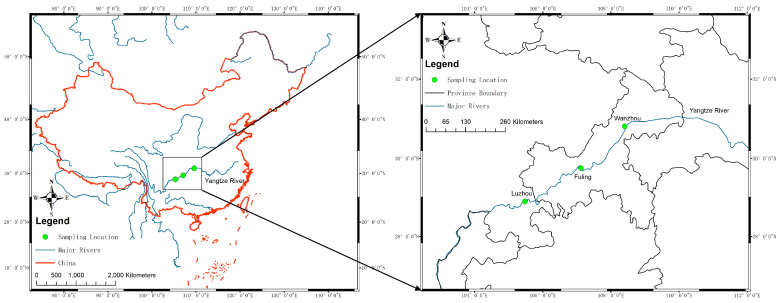
Sampling locations in the upper reaches of the Yangtze River. The sampling locations are indicated by green dots and labeled with city names. The blue line indicates the major rivers in China. The frame of China was labeled as a red line.

To investigate the viral communities in yellow catfish from the upper reaches of the Yangtze River, nine kidney tissue samples were collected from three sampling spots along the Yangtze River (LZ, FL, and WZ; Figure 1). Virome libraries were assembled and sequenced using the Illumina HiSeq platform after undergoing quality control. The libraries from these sites generated a total of 6,342,170 raw reads, with an average length of 200 bp. Reads were classified as eukaryotic and prokaryotic. Those with no significant similarity to any amino acid sequences in the NCBI database were discarded. 

### 3.2. Microbial Composition in the Upper Reaches of the Yangtze River

**Figure 2:**
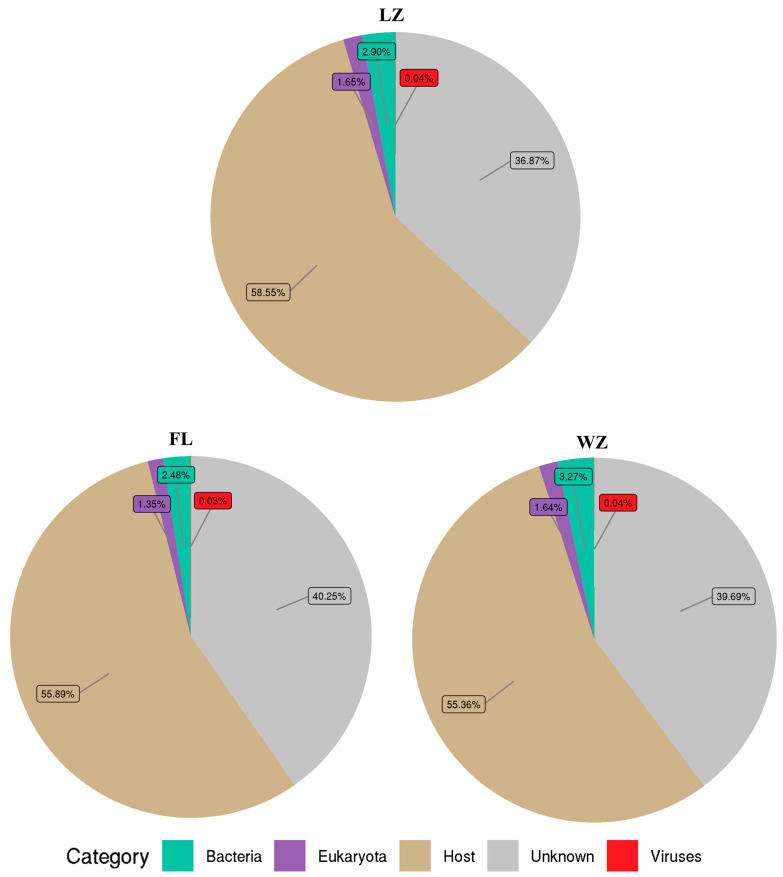
Microbial composition in the upper reaches of the Yangtze River. The relative abundance of the virome reads was categorized into various taxonomic groups based on the results of a BLASTx similarity search against the nr database. Reads that did not yield significant hits were classified as unknown. FL, Fuling; LZ, Luzhou; WZ, Wanzhou.

Among the remaining reads, 59.75–63.14% of the reads from tissue samples in the three regions were significantly similar to sequences deposited in the nr database. These reads were subsequently categorized into bacteria, viruses, eukaryotes, and cellular debris from the host (Figure 2). Viral sequences accounted for 0.03–0.04% of the total reads, and bacterial sequences for 2.48–3.27%. Meanwhile, sequences of eukaryota comprised 1.35–1.64%. Some of the sequences (36.87–40.25%) obtained in the viromes were unknown. 

For further taxon classification of yellow catfish in the three sampling sites, BLASTx analysis of the virome reads was conducted against a locally assembled virus database. As a result, the numbers of reads were assigned to viruses: LZ (0.04%), FL (0.03%), and WZ (0.04%) (Figure 2). Meanwhile, between 20.3% and 47.5% of the amino acids in these virus-related sequences aligned with predicted viral proteins from the NCBI database, suggesting a variety of unique viruses are present in these three regions.

### 3.3. Taxonomic Composition of the Viromes

**Figure 3:**
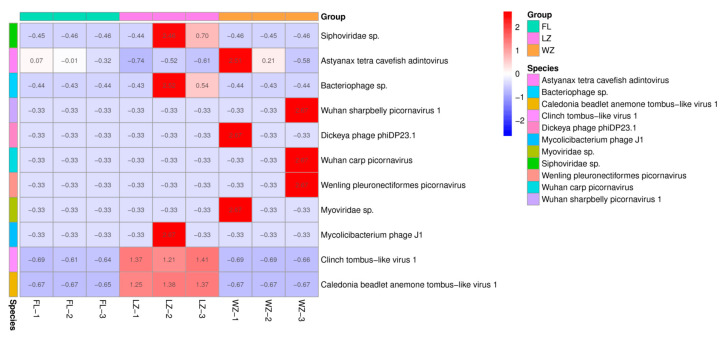
Taxonomic analyses of the viral read composition in three different regions in the upper reaches of the Yangtze River, including the taxonomic composition of the sequences at the viral species level. The sampling sites of FL, LZ, and WZ are indicated by green, pink, and orange rectangular strips and labeled with sampling names at the bottom.

The relative abundances of 11 viral species in the pooled samples from the three cities were determined by normalizing the sequence reads, as illustrated in Figure 3. Excluding reads that could not be classified into recognized families, the majority of reads within these three viromes were categorized into four dsDNA viral species, two ssDNA viral species, and five ssRNA viral species. The results show that the abundance of the 11 viruses varied widely across the three regions. Viral reads from the dsDNA family *Adintoviridae* were mainly distributed in FL of the upper reaches of the Yangtze River. Only a small number of sequences were categorized into other ssDNA families, including *Microviridae*. The most predominant virus family of dsDNA viruses was a bacteriophage family, *Siphoviridae*, in LZ. The virome from WZ contained the predominant order/family, including *Picornavirales*, *Caudovirales*, and *Adintoviridae*. Numerous sequence reads associated with mammalian viruses exhibited low nucleotide (nt) and amino acid (aa) sequence identity compared with known viruses. According to the genus and species classification criteria established by the International Committee on Taxonomy of Viruses for each viral family, these viruses could potentially represent 11 new species.

### 3.4. Viral Species Abundance in Three Regions

**Figure 4:**
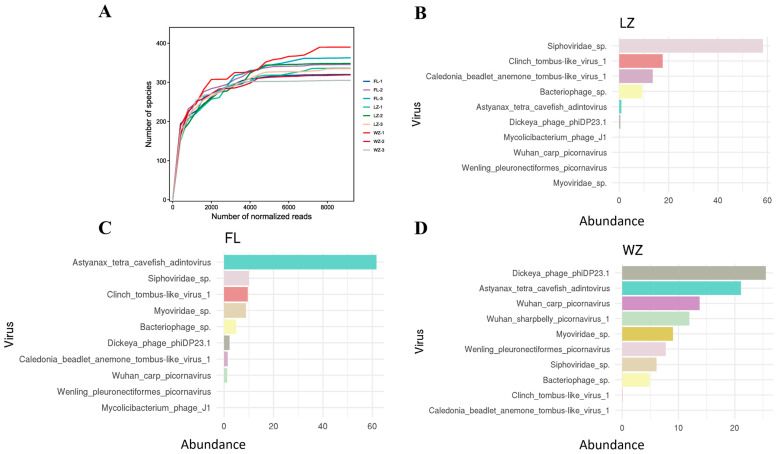
Rarefaction curves and the top 10 most abundant viral species in the three viromes in the upper reaches of the Yangtze River. (**A**) Rarefaction curves pertaining to viral species in each specific sample; (**B**–**D**) the ten most abundant viral species in the three viromes (FL, LZ, and WZ) from the upper reaches of the Yangtze River are presented. Shared species across each virome are marked with consistent colors. The legend on the *X*-axis represents the proportion of reads for these top 10 species relative to all reads assigned to viruses.

The rarefaction curves for the nine virome libraries showed a horizontal asymptote, indicating that the sequencing depth was adequate to detect almost all known viral species present in the samples, thus validating that the sequencing data were reliable and consistent (Figure 4A). Assessment of viral species diversity revealed the ten most prevalent viral species in each community (Figure 4B–D), with *Caudovirales*, *Adintoviridae*, and *Microviridae* being predominant in LZ, FL, and WZ, respectively.

Two groups of freshwater phage species were shared among all three libraries, *Bacteriophage* sp. and *Sipnoviridae* sp., which accounted for a large proportion of the viral species in each library. Additionally, certain viromes also exhibited unique species. For example, the sample from WZ contained the highest number of distinct viral species, including Wenling pleuronectiformes picornavirus, indicating that WZ had a distinctive viral community signature (Figure 4D).

### 3.5. Detection of Viruses in Yellow Catfish

**Figure 5:**
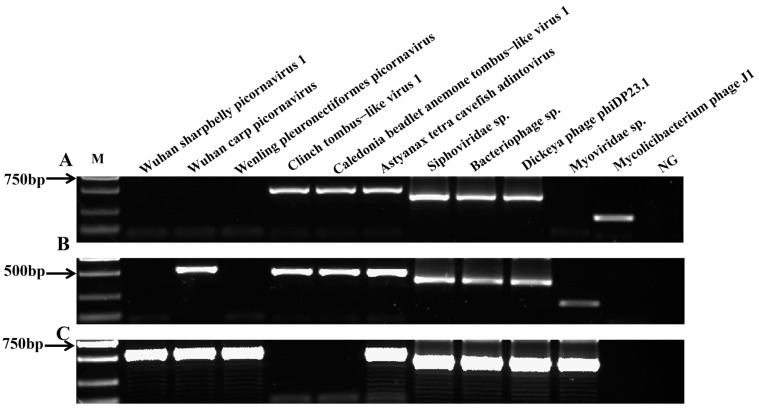
PCR detection of virus genes from yellow catfish in three sample regions. Agarose gel electrophoresis of virus genes from the yellow catfish kidney tissues in (**A**) LZ, (**B**) FL, and (**C**) WZ; M: DNA ladder (DL2000 bp); lane 1: Wuhan sharpbelly picornavirus 1; lane 2: Wuhan carp picornavirus; lane 3: Wenling pleuronectiformes picornavirus; lane 4: Clinch tombus-like virus 1; lane 5: Caledonia beadlet anemone tombus-like virus 1; lane 6: Astyanax tetra cavefish adintovirus; lane 7: *Siphoviridae* sp.; lane 8: *Bacteriophage* sp.; lane 9: Dickeya phage phiDP23.1; lane 10: *Myoviridae* sp.; lane 11: Mycolicibacterium phage J1; lane 12: negative control (NG).

The RT-PCR profiles of 11 virus genes were screened in the three regions in this study. In the LZ sampling site, 7 of 11 virus species were positive in the yellow catfish kidney samples, including Dickeya phage phiDP23.1, *Siphoviridae* sp., *Bacteriophage* sp., Mycolicibacterium phage J1, Clinch tombus-like virus 1, Caledonia beadlet anemone tombus-like virus 1, and Astyanax tetra cavefish adintovirus (Figure 5A). The results indicate the presence of Dickeya phage phiDP23.1, *Siphoviridae* sp., *Bacteriophage* sp., *Myoviridae* sp., Wuhan carp picornavirus, Clinch tombus-like virus 1, Caledonia beadlet anemone tombus-like virus 1, and Astyanax tetra cavefish adintovirus genes in the yellow catfish kidney samples from the FL site (Figure 5B). However, eight viruses were detected in the kidney tissue of yellow catfish from the WZ sampling sites (Dickeya phage phiDP23.1, *Siphoviridae* sp., *Bacteriophage* sp., *Myoviridae* sp., Wuhan carp picornavirus, Wuhan sharpbelly picornavirus 1, Wenling pleuronectiformes picornavirus, and Astyanax tetra cavefish adintovirus; Figure 5C). In addition, the positive PCR results of the above three sampling sites were further verified by sequencing, and the results show that the above amplified positive bands were the correct viral target species. 

### 3.6. Viral Diversity and Evolution

**Figure 6:**
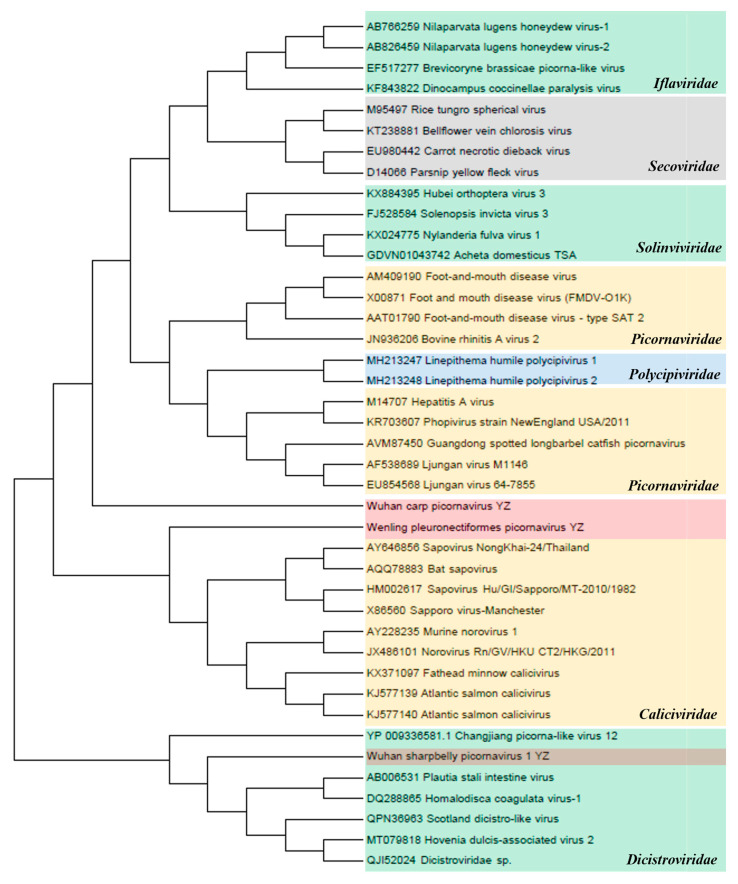
Phylogenetic analysis of the order *Picornavirales*. Phylogenetic analysis based on amino acid sequences of non-structural proteins in the order *Picornavirales*. The maximum-likelihood method was used to construct the phylogenetic trees with 1000 bootstraps from seven representative families. The newly identified viruses Wenling pleuronectiformes picornavirus YZ, Wuhan carp picornavirus YZ, and Wuhan sharpbelly picornavirus 1 YZ in this study are represented by shades of pink.

*Picornavirales*. The amino acid sequences of non-structural (NS) proteins in seven families of the order *Picornavirales* (*Caliciviridae*, *Dicistroviridae*, *Iflaviridae*, *Picornaviridae*, *Polycipiviridae*, *Secoviridae*, and *Solinviviridae*) were used for the phylogenetic analysis (Figure 6). The inferred NS proteins of Wuhan carp picornavirus, Wenling pleuronectiformes picornavirus, and Wuhan sharpbelly picornavirus 1 from the three sampling regions in the upper reaches of the Yangtze River (YZ) contained 22.3–38.4% similarity with the NS proteins of *Picornaviridae*, *Caliciviridae*, and *Dicistroviridae*, suggesting the existence of a new virus genus that has not been classified as *Picornavirales*. Phylogenetic analysis showed that Wuhan Carp picornavirus was close to the *Picornaviridae* family. However, Wenling pleuronectiformes picornavirus and Wuhan sharpbelly picornavirus 1 were close to *Caliciviridae* and *Dicistroviridae* viruses, respectively, according to the NCBI BLASTP analysis.

**Figure 7:**
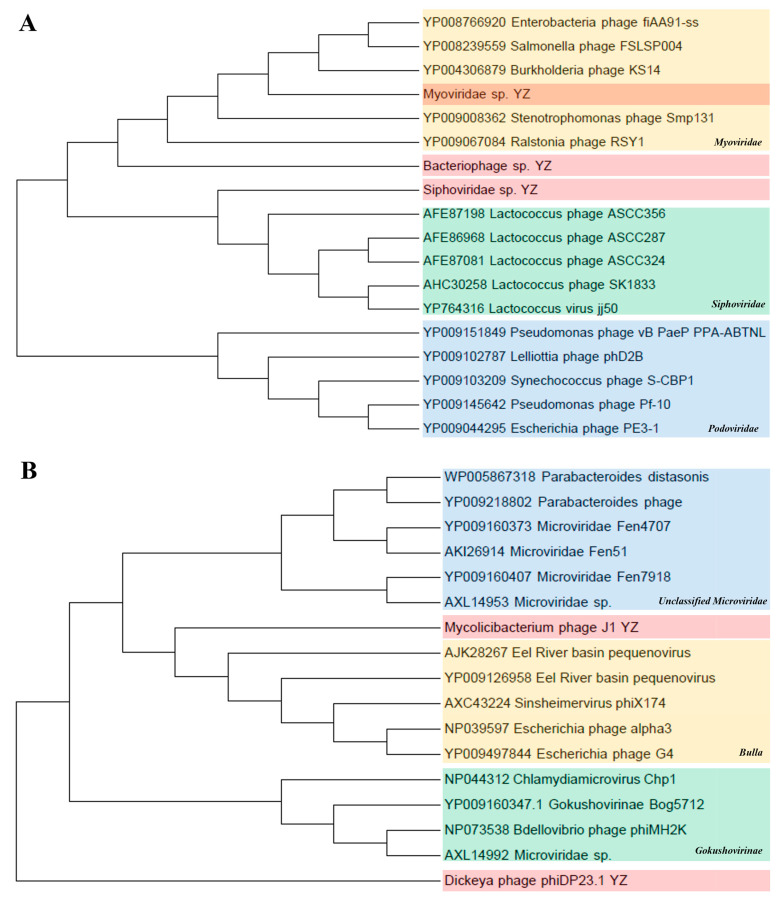
Phylogenetic analyses of *Caudovirales* and *Microviridae*. (**A**) Phylogenetic analysis of *Caudovirales* based on amino acid sequences of large terminase subunits (TerL). The maximum-likelihood method was used to construct the phylogenetic trees with 1000 bootstraps from 3 representative families. The newly identified viruses Bacteriophage sp., *Myoviridae* sp., and *Siphoviridae* sp. are represented by shades of pink. (**B**) Phylogenetic analysis of *Microviridae* based on the major capsid protein (MCP) amino acid sequence. Using the maximum-likelihood method, 1000 bootstrap samples from 3 representative families were selected to construct the phylogenetic trees. The newly discovered Mycobacterium phage J1 and the Dickeya phage phiDP23.1 are shown in pink.

*Caudovirales and Microviridae*. To further assess the commonality and diversity of the phage viral sequences identified in this study, we conducted phylogenetic analyses using the amino acid sequences from each representative complete hallmark gene region. *Caudovirales* comprises a group of dsDNA phages characterized by conserved regions in their large terminase subunits (TerL). Therefore, a phylogenetic tree was generated in this study based on the TerL protein sequences. The results show that the sequences of *Myoviridae* sp. and *Siphoviridae* sp. were close to the families of *Myoviridae* and *Siphoviridae*, respectively; however, *Bacteriophage* sp. formed a different independent branch in the order *Caudovirales* in the upper reaches of the Yangtze River, indicating that caudate bacteriophages had rich genetic diversity (Figure 7A). In addition, the major capsid protein (MCP) aa tree of the family *Microviridae* showed that the sequences of Mycobacterium phage J1 and Dickeya phage phiDP23.1 were phylogenetically attributed to the *Bulla* lineage and *Gokushovirinae* lineage, respectively (Figure 7B).

**Figure 8:**
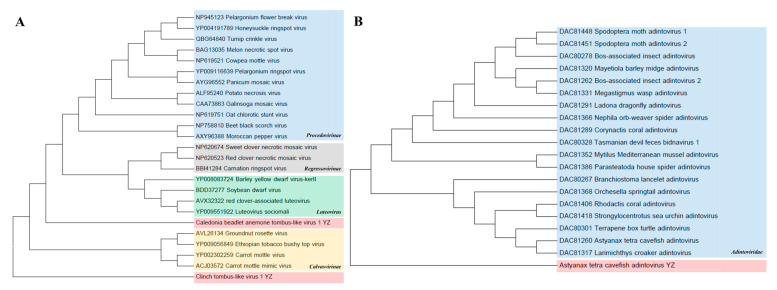
Phylogenetic analyses of *Tombusviridae* and *Adintoviridae*. (**A**) Phylogenetic analysis of *Tombusviridae* based on RNA-dependent RNA polymerase (RdRp) amino acid sequence. Using the maximum-likelihood method, 1000 bootstrap samples from 4 representative families were selected to construct the phylogenetic trees. The newly discovered Caledonian cephalanthus Tombus-like virus 1 and Clinch Tombus-like virus 1 are shown in pink. (**B**) Phylogenetic analysis of *Adintoviridae* based on type B DNA polymerase (PolB) amino acid sequence. The newly discovered Astyanax tetra cavefish adenovirus is shown in pink.

*Tombusviridae*. Two Tombus-like virus species, Caledonian Cephaloanthus Tombus-like virus 1 and Clinch Tombus-like virus 1, were identified in this study and belong to the *Lutevirus* and *Calvusvirinae* groups, which are related to *Tombusviridae*. In the phylogenetic tree of amino acid RNA-dependent RNA polymerase (RdRp), Caledonian Cephalodonia tombus-like virus 1 was clustered with *Lutevirus*, with homology ranging from 37.9 to 47.2%. The homology between Clinch tombus-like virus 1 and the *Calvusvirinae* group was 33.6–42.3% (Figure 8A).

*Adintoviridae*. A phylogenetic tree was constructed based on type B DNA polymerase (PolB) amino acid sequences. The results show that the Astyanax tetra cavefish adintovirus identified here was clustered into the family *Adintoviridae* and formed a separate clustered branch. This virus is a novel virus, showing amino acid identities of 28.2–34.3% with the family *Adintoviridae* (Figure 8B).

## 4. Discussion 

Aquatic ecosystems harbor a diverse array of viruses that play a pivotal role in regulating bacterial communities and influencing biogeochemical cycles [27]. Furthermore, numerous viruses can induce mass mortality in aquatic organisms, thereby posing significant threats to the sustainable development of aquaculture [1,2,3]. However, research on viral communities within river systems remains limited. In this study, representative fish, yellow catfish, were selected from three sampling sites, and RNA-seq analysis was conducted to determine the genetic diversity of viruses in the upper reaches of the Yangtze River. Comparative analysis of these virome sequences against the nr database revealed that 59.75–63.14% of reads from tissue samples across the three regions exhibited significant similarity to entries in the nr database. These sequences were further categorized into viral, bacterial, eukaryotic, and host cell fragments. Notably, most sequences with substantial matches among pathogenic microorganisms corresponded to bacterial sequences. Comparable findings were observed in the investigation of viral community distributions within the surface waters of the East China Sea and the Yangtze River [10,28]. This discrepancy might be attributed to the considerably larger size of bacterial genomes compared with those of viruses, resulting in a greater number of read sequences generated during sequencing for bacteria than for viruses, thereby increasing their relative abundance within the samples [10]. 

In this study, the composition and abundance of the viral community in the three regions within the upper reaches of the Yangtze River exhibited notable regional variations. The predominant viral families/order identified were *Caudovirales*, *Adinoviridae*, and *Microviridae* in LZ, FL, and WZ, respectively. A substantial number of phage sequences were detected across these three sites, encompassing *Siphoviridae*, *Myoviridae*, *Microviridae*, and other phage families, most of which belong to the order *Caudovirales*. These findings indicate that caudate bacteriophages are dominant within the known viral groups when compared with eukaryotic DNA viruses [29]. Furthermore, our results demonstrate a numerical predominance of bacteriophages in the upper reaches of the Yangtze River, which is consistent with previous reports on freshwater virus communities from regions such as the East Lake and the Jiulong River estuary in China [7,30]. Given that *Caudovirales* currently dominate available phage sequence databases, there is an increased likelihood of matching members from this order over any other phage group. Additionally, the extensive representation of phage genomes within these databases facilitates the easier assignment of query sequences to this order; thus, it is reasonable that the order *Caudovirales* typically constitutes a significant proportion of identified phages.

Species-level analysis revealed that nearly all known viral species present in our samples were captured via RNA-seq, without significant differences observed among sample species counts. However, only 11 viral sequences were discerned in our investigations; this relatively low yield contrasts sharply with findings reported elsewhere [7,10,28,30]. We hypothesized that this discrepancy might stem from our focus on samples from yellow catfish, while other high-throughput sequencing efforts predominantly utilized water samples derived from river ecosystems where concentrations and diversity of virions are likely to be higher than those found within fish tissues following post-concentration processes. Similarly, the underrepresentation of freshwater viruses within public databases concerning detection rates revealed only 11 unique viruses across three distinct communities, suggesting a vast reservoir of undiscovered viral entities that might persist within yellow catfish populations inhabiting these upper river stretches. Moreover, whether the deficiencies noted regarding bacteriophage-related sequences associated with yellow catfish’s virome can be attributed to methodological limitations inherent to sequencing approaches, sampling bias, or perhaps even unidentified factors warrants further exploration.

The analysis of the viral community composition revealed that LZ, FL, and WZ shared two identical viruses, and this finding is geographically consistent with the three sampling locations, being particularly influenced by the dilution effects of the Yangtze River water. However, it is essential to acknowledge the differences in the viral communities between these three sampling sites, which might reflect variations in the ecological habitats between them. For instance, sample sites from LZ were situated far from urban areas and exhibited minimal contamination from human activities. Consequently, this region maintains high primary productivity and a robust availability of microbial hosts, such as bacteriophages primarily derived from bacteria and plant-associated viruses (tombus-like viruses). However, it harbors relatively few virus species overall. Notably, the abundance of picornaviruses in WZ exhibited a distinct pattern within its viral community structure. Most of the unique viral species identified in our study were located in WZ. Among them, Wenling pleuronectiformes picornavirus has been shown to infect vertebrates [31], and *Picornaviridae* constitutes a substantial family of small, positive-sense single-stranded RNA viruses that are responsible for a range of significant diseases in both humans and animals [32]. The observed differences in the viral community structures might be attributed to WZ being the only provincial capital among the three sampling sites, comprising a densely populated area with a comparatively fragile ecological environment that is potentially impacted by municipal and industrial wastewater discharge, which could result in unique hydrological characteristics influencing its distinctive viral composition. 

Furthermore, a highly contagious disease affecting yellow catfish emerged in farmed populations in 2020, posing a significant threat to the sustainable development of the yellow catfish aquaculture industry in China. The pathogen responsible for this disease has been recognized as a novel *Calicivirus*, provisionally designated as yellow catfish calicivirus (YcCV) [18]. In this study, three species of picornaviruses were obtained from the WZ sampling site: Wenling pleuronectiformes picornavirus, Wuhan carp picornavirus, and Wuhan sharpbelly picornavirus 1. Notably, Wenling pleuronectiformes picornavirus is clustered within *Caliciviridae*. However, upon examining the sequence homology between Wenling pleuronectiformes picornavirus and YcCV viruses, we determined that there was no nucleotide sequence homology between the two viral sequences, whereas approximately 30% similarity was observed at the amino acid level for their NS proteins. Additionally, the parental stock of yellow catfish primarily comprises wild specimens collected from the Yangtze River Basin or adjacent lake areas for breeding purposes [23]. Therefore, further evolutionary studies are warranted to determine whether the viral pathogen affecting industrial yellow catfish arose from evolutionary mutations of picornaviruses endemic to the Yangtze River basin. These findings also provide valuable reference data to trace the source of YcCV pathogens associated with yellow catfish. 

## 5. Conclusions

This study represents the first investigation of the viral community and diversity associated with yellow catfish in the upper reaches of the Yangtze River. The results reveal a substantial presence of various viruses, including *Adintoviridae*, *Tombusviridae*, *Caudovirales*, *Microviridae*, *Picornavirales*, and other bacteriophage families, many of which are reported for the first time. The virome from WZ exhibited a distinct viral community composition, showing a relatively high abundance of the order *Picornavirales* in comparison with the other two sampling regions. In LZ, the most dominant family among dsDNA viruses was the bacteriophage family *Siphoviridae*. Despite certain limitations regarding sample size and sequencing methodologies, this research significantly enhances our understanding of yellow catfish-associated viruses in the Yangtze River and encourages further exploration into the diversity of viral species from a broader perspective. The advanced technologies employed herein provide a theoretical foundation for subsequent investigations into pathogen transmission dynamics and strategies to prevent viral diseases affecting yellow catfish. Our results also provide support for the diagnosis and treatment of infectious diseases prevalent in the upper reaches of the Yangtze River.

## Figures and Tables

**Table 1:** Origins of the yellow catfish sampled from different regions in this study.

Sampling Location	Abbreviation	City	Date of Collection	Temperature (°C)	Size (cm)
Luzhou-1	LZ-1	Luzhou	23 July 2023	21	14
Luzhou-2	LZ-2		24 July 2023	22	17
Luzhou-3	LZ-3		25 July 2023	21	16
Fuling-1	FL-1	Fuling	12 June 2023	22	15
Fuling-2	FL-2		12 June 2023	21	18
Fuling-3	FL-3		13 June 2023	23	16
Wanzhou-1	WZ-1	Wanzhou	4 July 2023	21	15
Wanzhou-2	WZ-2		5 July 2023	23	14
Wanzhou-3	WZ-3		6 July 2023	21	15

**Table 2:** Primer sequences used for PCR amplification of virus genes in yellow catfish.

Gene Name	Primer Name	Primers (5′-3′)	Product Size (bp)
Wuhan sharpbelly picornavirus 1	WSPV-F	TTCACAGGTCTCGGACACTGCCAAT	542
	WSPV-R	TCATGACAGTGACAGTGTTTTCTAC	
Wuhan carp picornavirus	WCPV-F	TACACTCGCATTCGTGGGATCAAG	551
	WCPV-R	TGTTTCGAATCGCATTAGTAACTCT	
Wenling pleuronectiformes picornavirus	WPPV-F	TTCGCCAGTTTTCTTTTGACCGCT	560
	WPPV-R	GCCATCAGCTCTTTATTGAGGTG	
Clinch tombus-like virus 1	CTV-F	TGTGGAAGTCGGTTGGTTTGGTTCT	537
	CTV-R	TAGATCCGTACCCGCGAACGGCGG	
Caledonia beadlet anemone tombus-like virus 1	CBTV-F	TATACATCTAGGTGCTCCATAGT	541
	CBTV-R	TGGTCGCACAGGGTGGTTTGTGAAAAC	
Astyanax tetra cavefish adintovirus	ATCV-F	TCATAGTGAACAACAACAACTGAT	542
	ATCV-R	TTATGTTGAAATACCTCTGCTTT	
*Siphoviridae* sp.	SP-F	TCACTCGAGCGCCTTAGTATATT	407
	SP-R	TAGGTATAAGTAACGATAAAGGGGGT	
*Bacteriophage* sp.	BS-F	GCGTCGTTATTTTGCAGAAGCAG	389
	BS-R	TCACCCTCGCCTAGCGGATTTTC	
Dickeya phage phiDP23.1	DP-F	TACACGGTGGATGCCTAGGCAGT	402
	DP-R	TGTACGGGGCTATCACCCTGTAT	
*Myoviridae* sp.	MP-F	CAGACAGTCGCCGCTGTGTCGT	405
	MP-R	TCGTCTAGGCCAGCAATCGCTC	
Mycolicibacterium phage J1	MPJ-F	TATTGAGAAAGACATAATGGTTAT	301
	MPJ-R	GCTTATACCACATGAAATACAGT	

## Data Availability

Data are contained within the article.

## References

[1] Korajkic A., Wanjugi P., Brooks L., Cao Y.P., Harwood V.J. (2019). Persistence and decay of fecal microbiota in aquatic habitats. Microbiol. Mol. Biol. Rev..

[2] Liu W.Z., Zhang Y.C., Ma J., Jiang N., Fan Y.D., Zhou Y., Cain K., Yi M.S., Jia K.T., Wen H. (2020). Determination of a novel parvovirus pathogen associated with massive mortality in adult tilapia. PLoS Pathog..

[3] Costa V.A., Holmes E.C. (2024). Diversity, evolution, and emergence of fish viruses. J. Virol..

[4] Kibenge F.S. (2019). Emerging viruses in aquaculture. Curr. Opin. Virol..

[5] Chen Y.M., Sadiq S., Tian J.H., Chen X., Lin X.D., Shen J.J., Chen H., Hao Z.Y., Wille M., Zhou Z.C. (2022). RNA viromes from terrestrial sites across China expand environmental viral diversity. Nat. Microbiol..

[6] Gregory A.C., Zayed A.A., Conceição-Neto N., Temperton B., Bolduc B., Alberti A., Ardyna M., Arkhipova K., Carmichael M., Cruaud C. (2019). Marine DNA Viral macro- and microdiversity from Pole to Pole. Cell.

[7] Ge X.Y., Wu Y.Q., Wang M.N., Wang J., Wu L.J., Yang X.L., Zhang Y.J., Shi Z.L. (2013). Viral metagenomics analysis of planktonic viruses in East Lake, Wuhan, China. Virol. Sin..

[8] Fernandez-Cassi X., Timoneda N., Martínez-Puchol S., Rusiñol M., Rodriguez-Manzano J., Figuerola N., Bofill-Mas S., Abril J.F., Girones R. (2018). Metagenomics for the study of viruses in urban sewage as a tool for public health surveillance. Sci. Total Environ..

[9] Kim Y., Gim A.T., Teal T.K., Rose J.B. (2015). Metagenomic investigation of viral communities in ballast water. Environ. Sci. Technol..

[10] Lu J., Yang S.X., Zhang X.D., Tang X.M., Zhang J., Wang X.C., Wang H., Shen Q., Zhang W. (2022). Metagenomic analysis of viral community in the Yangtze River expands known eukaryotic and prokaryotic virus diversity in freshwater. Virol. Sin..

[11] Djikeng A., Kuzmickas R., Anderson N.G., Spiro D.J. (2009). Metagenomic analysis of RNA viruses in a fresh water lake. PLoS ONE.

[12] Jo W.K., Osterhaus A.D., Ludlow M. (2018). Transmission of morbilliviruses within and among marine mammal species. Curr. Opin. Virol..

[13] Du H., Wu J., Zeng S.D., Xia J. (2023). Historical attributions and future projections of gross primary productivity in the Yangtze River Basin under climate change based on a novel coupled LUE-RE model. Remote Sens..

[14] Chen Z.Y., Li J.F., Shen H.T., Wang Z.H. (2001). Yangtze River of China: Historical analysis of discharge variability and sediment flux. Geomorphology.

[15] Feng H.P., Kang P., Deng Z.C., Zhao W., Hua M., Zhu X.Y., Wang Z. (2023). The impact of climate change and human activities to vegetation carbon sequestration variation in Sichuan and Chongqing. Environ. Res..

[16] Liu Y., Wu P.D., Zhang D.Z., Zhang H.B., Tang B.P., Liu Q.N., Dai L.S. (2019). Mitochondrial genome of the yellow catfish *Pelteobagrus fulvidraco* and insights into *Bagridae* phylogenetics. Genomics.

[17] Guo W.J., Guo C.T., Wang Y.H., Hu W.H., Mei J. (2019). Population structure and genetic diversity in yellow catfish (*Pelteobagrus fulvidraco*) assessed with microsatellites. J. Genet..

[18] Liu W.Z., Xue M.Y., Yang T., Li Y.Q., Jiang N., Fan Y.D., Meng Y., Luo X.W., Zhou Y., Zeng L.B. (2022). Characterization of a Novel RNA Virus Causing Massive Mortality in Yellow Catfish, *Pelteobagrus fulvidraco*, as an Emerging Genus in *Caliciviridae* (*Picornavirales*). Microbiol. Spectr..

[19] Ye S.G., Li H., Qiao G., Li Z.S. (2009). First case of *Edwardsiella ictaluri* infection in China farmed yellow catfish *Pelteobagrus fulvidraco*. Aquaculture.

[20] Zhou Y., Jiang N., Zeng J., Fan Y.D., Liu W.Z., Si K.G., Zeng L.B. (2019). Isolation and identification of pathogenic bacterium from ascites disease of yellow catfish, *Pelteobagrus fulvidraco*. Chin. Fish. Qual. Stand..

[21] Li W.X., Wang G.T., Yao W.J., Nie P. (2010). Frequency distribution and seasonal dynamics of intestinal helminths in the yellow head catfish *Pelteobagrus fulvidraco* from Liangzi Lake, China. Comp. Parasitol..

[22] Zhang X.D., Shen W.Y., Xu C.C., Wang Y.D., Xu H., Liu X.Y., Wei Y.W. (2019). Discovery of a novel Piscanivirus in yellow catfish (*Pelteobagrus fulvidraco*) in China. Infect. Genet. Evol..

[23] Xiao M.S., Bao F.Y., Cui F. (2014). Pattern of genetic variation of yellow catfish *Pelteobagrus fulvidraco* Richardso in Huaihe river and the Yangtze River revealed using mitochondrial DNA control region sequences. Mol. Biol. Rep..

[24] Fang D.A., Yang X.J., Zhou Y.F., Xu D.P., Yang Y., Qin C.J. (2020). Discovery of the indicator role of period 2 in yellow catfish (*Pelteobagrus fulvidraco*) food intake during early life development stages. Chronobiol. Int..

[25] Chen Q.L., Luo Z., Liu C.X., Zheng J.L., Zhu Q.L., Hu W., Zhuo M.Q. (2015). Effects of waterborne copper exposure on carnitine composition, kinetics of carnitine palmitoyltransferases I (CPT I) and mRNA levels of CPT I isoforms in yellow catfish *Pelteobagrus fulvidraco*. Chemosphere.

[26] Zhong L.Q., Song C., Wang M.H., Chen Y.M., Qin Q., Pan J.L., Chen X.H. (2013). Genetic diversity and population structure of yellow catfish *Pelteobagrus fulvidraco* from five lakes in the middle and lower reaches of the Yangtze River, China, based on mitochondrial DNA control region. Mitochondrial DNA.

[27] Jover L.F., Effler C., Buchan A., Wilhelm S.W., Weitz J.S. (2014). The elemental composition of virus particles: Implications for marine biogeochemical cycles. Nat. Rev. Microbiol..

[28] Wu S., Zhou L., Zhou Y.F., Wang H.M., Xiao J.Z., Yan S.L., Wang Y.J. (2020). Diverse and unique viruses discovered in the surface water of the East China Sea. BMC Genom..

[29] Koonin E.V., Dolja V.V., Krupovic M. (2015). Origins and evolution of viruses of eukaryotes: The ultimate modularity. Virology.

[30] Cai L.L., Zhang R., He Y., Feng X.Y., Jiao N.Z. (2016). Metagenomic Analysis of virioplankton of the subtropical Jiulong River Estuary, China. Viruses.

[31] Hargitai R., Pankovics P., Boros A., Mátics R., Altan E., Delwart E., Reuter G. (2021). Novel picornavirus (family *Picornaviridae*) from freshwater fishes (*Perca fluviatilis*, *Sander lucioperca*, and *Ameiurus melas*) in Hungary. Arch. Virol..

[32] Jiang P., Liu Y., Ma H.C., Paul A.V., Wimmer E. (2014). Picornavirus morphogenesis. Microbiol. Mol. Biol. Rev..

